# Successful management of anastomotic false aneurysm after a femoro-popliteal bypass with percutaneous injection of fibrin sealant

**DOI:** 10.1590/1677-5449.20250241

**Published:** 2026-08-28

**Authors:** Thilina Gunawardena, Nuwan Anupama, Feros Mohomed

**Affiliations:** 1 Teaching Hospital, Kurunegala, Sri Lanka.

**Keywords:** anastomotic false aneurysm, vein bypass, percutaneous thrombin, pseudoaneurisma anastomótico, bypass, trombina percutânea

## Abstract

Anastomotic false aneurysms are rare after autologous vein bypass. However, such lesions have the potential for life or limb-threatening complications, and their management can be extremely challenging. Here, we describe a 62-year-old male patient who developed a false aneurysm at the proximal anastomosis after a femoro-popliteal bypass. His left upper limb cephalic vein had been used as the conduit. The false aneurysm was successfully managed by percutaneous injection of fibrin sealant. This report aims to highlight this rare complication and its successful percutaneous management, with preservation of the bypass graft.

## Resumo

Pseudoaneurismas anastomóticos são raros após *bypass* venoso autólogo. No entanto, tais lesões têm potencial para desenvolver complicações que ameaçam a vida ou os membros do paciente, e seu manejo pode ser extremamente desafiador. Neste estudo, descrevemos o caso de um paciente do sexo masculino, 62 anos, que desenvolveu pseudoaneurisma na anastomose proximal após *bypass* femoropoplíteo. A veia cefálica do membro superior esquerdo foi usada como conduto. O pseudoaneurisma foi tratado com sucesso por injeção percutânea de selante de fibrina. Este relato tem como objetivo destacar tal complicação rara e seu manejo percutâneo bem-sucedido, com preservação do enxerto de *bypass*.

## INTRODUCTION

Anastomotic false aneurysms occurring after arterial reconstructions using autologous veins are rare, but can cause significant morbidity and occasional mortality.^1^ Technical errors, challenging anastomoses in calcified arteries, and infection have been identified as possible etiologies for such false aneurysms during the early postoperative period. Late anastomotic false aneurysms are usually a result of vessel wall degeneration from progressive atherosclerosis^1,2^ Kang et al.^3^ described ultrasound-guided thrombin injection as a successful alternative to traditional open repair of post-catheterization femoral false aneurysms. Since this method was described, its utilization has expanded to include false aneurysms from other causes as well.^4^ Here, we describe a patient who developed a false aneurysm at the proximal anastomosis after a femoro-popliteal bypass. Rather than opting for open surgical revision, we were able to successfully manage the lesion percutaneously using ultrasound-guided fibrin sealant injection.

Informed written consent was obtained from the patient before data collection and publication of the article. The study was done according to the standards of the institutional ethics committee and the declaration of Helsinki.

### Part 1 - Clinical case

A 63-year-old male with a history of type 2 diabetes mellitus, hypertension, and stage 3 chronic kidney disease (CKD) was referred to the vascular surgical unit at the teaching hospital in Kurunegala, Sri Lanka, with a necrotic left big toe. He was an ex-smoker and had undergone percutaneous coronary stenting following a non-ST elevation myocardial infarction 2 years back. On examination, his left popliteal and pedal pulses were absent with a good volume femoral pulse. The ankle brachial pressure index on the left was 1.5, indicative of vessel wall calcification. A long segment occlusion involving the mid to distal superficial femoral artery, a stenosed infragenicular popliteal artery (PA), and preserved anterior tibial artery (ATA) run-off to the foot were noted on duplex ultrasound (DUS). Vein mapping of the lower limbs showed sub-optimal saphenous veins for a native vein bypass, but the cephalic vein in the left upper limb was of adequate caliber. With reasonable exercise tolerance and preserved left ventricular ejection fraction of 60% on the 2D echocardiogram, we decided to proceed with a femoral-to-infragenicular popliteal artery bypass using the left cephalic vein.

**Figure 1:**
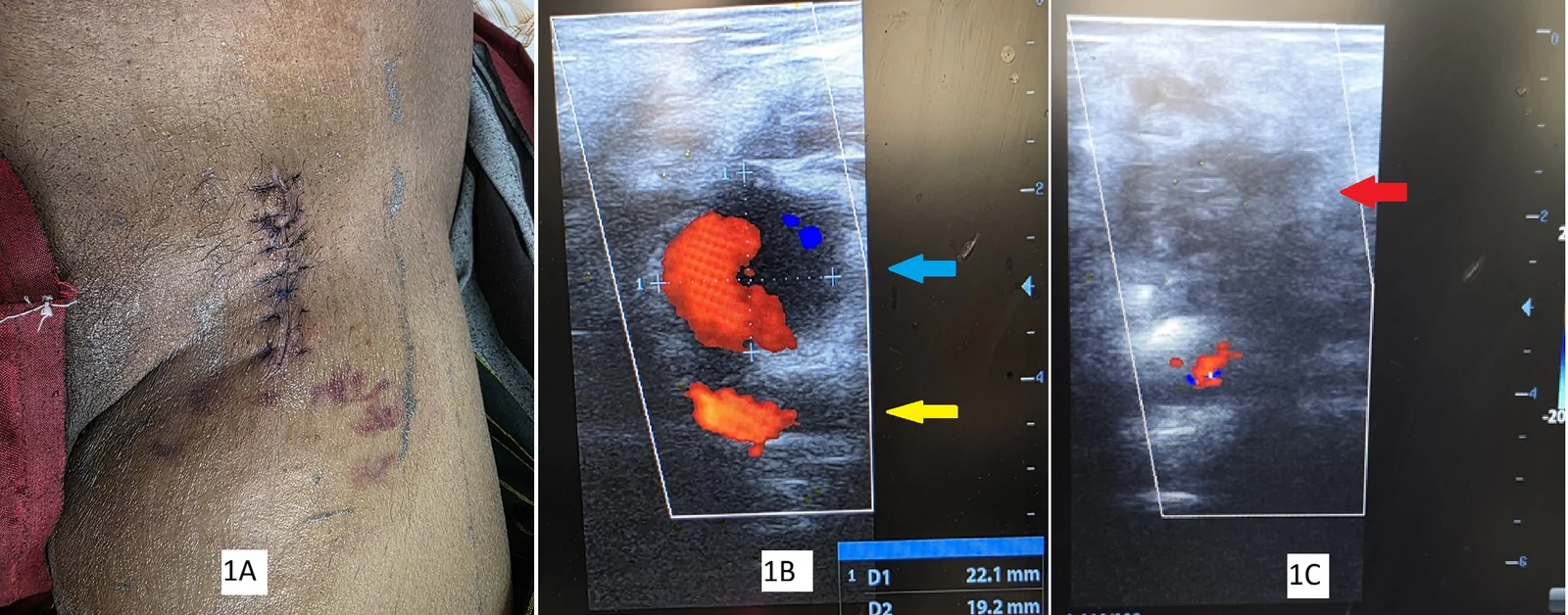
(A) Groin incision without features of infection; (B) False aneurysm on duplex ultrasound (Blue arrow: aneurysm, Yellow arrow: vein graft); (C) Thrombosed false aneurysm after percutaneous fibrin sealant injection (Red arrow).

The surgery was done under general anesthesia. The left common femoral artery (CFA) was exposed with a longitudinal incision. The medial approach was used to access the infragenicular popliteal artery, which was dissected up to the origin of the ATA. The left cephalic vein was harvested and reversed. The proximal anastomosis was performed end-to-side to the CFA using a continuous 6 0 polypropylene suture, and the graft was tunneled subfascially. The distal anastomosis was to the infragenicular PA, directed towards the origin of the ATA, using a continuous 6 0 polypropylene suture in an end-to-side fashion. After reperfusion, there was a palpable dorsalis pedis pulse, and the necrotic toe was amputated. According to the wound tissue culture sensitivity, he was started on intravenous (IV) piperacillin, which was continued for 7 days. He was discharged on post op day 7 but readmitted 3 days later due to sudden bleeding from his groin wound. The bleeding settled following application of direct, manual pressure. The groin incision did not show clinical features of inflammation (Figure 1A), but his white cell count (16 x 10^3^ mm^3^) and C-reactive protein level (212 mg/dL) were elevated. A DUS of the groin revealed a false aneurysm measuring 22.1 x 19.2 mm arising from the proximal anastomosis (Figure 1B).

### Part 2 - What was done

Due to the raised inflammatory markers, we suspected subclinical groin infection and started him on empirical antibiotic therapy with IV meropenem and IV vancomycin. Blood cultures were taken before initiation of antibiotics, but they were negative. To treat the false aneurysm and to preserve the bypass graft, we decided to proceed with percutaneous fibrin sealant injection.

Under ultrasound guidance, fibrin sealant was slowly injected into the false aneurysm using an 18-gauge needle, achieving complete thrombosis of the lesion (Figure 1C). We used 2 mL of fibrin sealant, a commercial preparation containing thrombin and fibrinogen (Tisseel®, Baxter AG, Vienna, Austria). The patient was continued on IV antibiotics until the inflammatory markers normalized and was discharged home with a working bypass graft. At the 3-month follow-up, the amputation site of the big toe has nearly healed (Figures 22B), and the bypass remains patent, with no recurrence of the false aneurysm.

**Figure 2:**
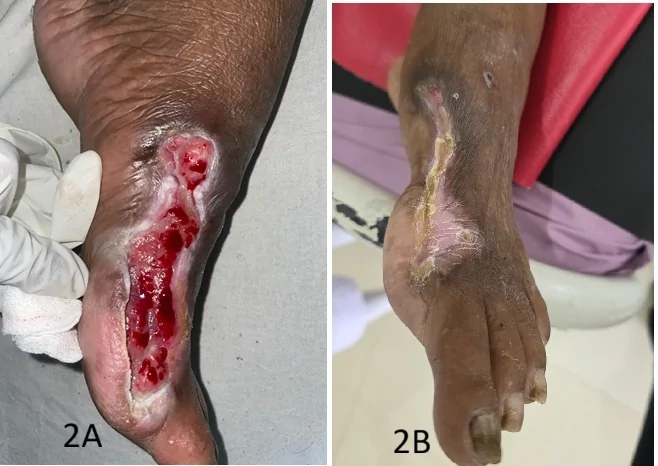
(A) Foot ulcer soon after the bypass; (B) Foot ulcer 3 months after the bypass.

## DISCUSSION

Anastomotic false aneurysms are an extremely rare complication after autologous vein bypasses, with a reported incidence of less than 1%.^2^ Although rare, they can be associated with severe consequences such as graft thrombosis, distal embolization, or even death from hemorrhage.^1^ Patients who develop this complication can present with bleeding, painful pulsatile masses, or thrombosed bypass grafts. Occasionally, asymptomatic patients may be detected on routine graft surveillance scans.^1,5^ Our patient presented with bleeding from the proximal anastomosis on postoperative day 10. We were able to control the bleeding with the application of direct pressure, so he did not require urgent re-exploration. However, patients with ongoing hemorrhage or features of acute limb ischemia due to graft thrombosis will require emergency procedures to preserve life or limb.^1^

Technical errors, grafting to calcified arteries, and infection have been reported as possible reasons for anastomotic false aneurysms in the early postoperative period.^1,2^ During the era when silk was commonly used as a suture material for vascular anastomoses, suture degeneration was also a culprit of such lesions.^2,6^ Endarterectomy at the site of the anastomosis can lead to false aneurysm formation, probably due to loss of arterial wall strength.^2^ Degeneration of the native artery due to progressive atherosclerosis has been cited as the main reason for late-onset false aneurysms.^1^ Although our patient had risk factors for medial vessel wall calcification, such as diabetes and CKD, his common femoral artery, which was used as the inflow for the bypass, was relatively disease-free. The anastomosis was performed with continuous 6 0 polypropylene suture, as per our standard operative procedure. When he presented with bleeding from his groin, the wound appeared to be completely healed without overt features of infection. His blood cultures were negative, but leukocytosis and elevated CRP were suggestive of an ongoing infective process. Considering all, we postulated that subclinical infection may be the most likely cause for the anastomotic false aneurysm in our patient.

Reoperation is the traditional approach to managing anastomotic false aneurysms after arterial reconstructions. Simple suture at the site of anastomotic disruption is not recommended due to the high risk of recurrence. So, ligation of the artery proximal and distal to the anastomosis, and revision of the bypass with a more proximal inflow would be required.^6^ Such operations can be technically challenging, and patients with compromised physiological reserves due to bleeding and multiple comorbidities may not tolerate such complex procedures. Studies that report outcomes after such re-operations, primarily for false aneurysms after aorto-femoral reconstructions, quote substantial procedure-related morbidity rates.^7^ In contrast, ligation of the artery and the graft without restoring vascular continuity has a high risk of major amputation. Endovascular stenting has been described as an option to treat selected patients with false aneurysms at the groin after aorto-femoral reconstructions.^8^ This option would have been unsuitable in our case due to the significant size mismatch between the CFA and the vein graft. Additionally, such stenting would exclude the profunda, risking severe limb ischemia in the event of graft thrombosis.

Since their introduction, percutaneous thrombin injections have been used successfully to treat post-catheterization false aneurysms of the femoral artery.^3^ With accumulating experience, the technique has been used to deal with false aneurysms due to other etiologies as well.^4^ Infrequently, this method has been used to treat post-anastomotic aneurysms, especially after open aortic surgery.^9^ However, the use of thrombin injection for false aneurysms after native vein bypass has rarely been documented. Our literature survey identified a single case report, in which Nakamura^5^ used this technique to treat a false aneurysm at the distal anastomosis after a femoro-peroneal bypass. Farrell et al.^10^ used thrombin injection to successfully treat a false aneurysm in a vein bypass that resulted from a slipped side branch ligature.

Thrombin, when injected into the false aneurysm, will trigger thrombosis and closure of the lesion. Theoretically, fibrin sealant, which contains both thrombin and fibrinogen, should be more efficacious in this process. However, this is contradicted by the findings of Pinto et al., who reported that thrombin achieved superior closure rates compared with fibrin sealant in managing post-catheterization femoral false aneurysms.^11^ In their study, large, complex lesions were preferentially treated with fibrin sealant, which may have contributed to inferior outcomes.

Management of false aneurysms with percutaneous injections can be complicated by thrombosis of the graft or the native artery and distal embolization. To prevent such events, precise positioning of the needle tip into the aneurysm, away from its neck, and injection of the drug in small increments have been suggested.^8^ Contraindications for this technique are the presence of overt infection, false aneurysms with wide necks, necrosis of the overlying skin, and pressure effects from the lesion.^5^

In our case, we opted for percutaneous fibrin sealant injection for several reasons. First, we wanted to avoid the morbidity of redo surgery. Additionally, if we were to revise the bypass, our patient lacked a suitable vein conduit, as we had exhausted his only option, the left upper limb cephalic vein. As we were suspecting subclinical infection as a possible etiology for the false aneurysm, use of a prosthetic graft would have been problematic. Our previous experience with reopening and closing the anastomotic gap at the site of the leak has not been good, with almost all progressing to rebleeding and ligation of the native artery, followed by limb loss and/or death.

## CONCLUSION

Although rare, an anastomotic false aneurysm after autologous vein bypass is a challenging complication. In selected cases, excellent outcomes can be achieved by percutaneous injection of thrombin or fibrin sealant to close these lesions with preservation of graft patency and minimal procedure-related morbidity.

## Data Availability

Data sharing does not apply to this article, as no data were generated or analyzed.
